# Protection of Poly(Vinyl Chloride) Films against Photodegradation Using Various Valsartan Tin Complexes

**DOI:** 10.3390/polym12040969

**Published:** 2020-04-21

**Authors:** Alaa Mohammed, Gamal A. El-Hiti, Emad Yousif, Ahmed A. Ahmed, Dina S. Ahmed, Mohammad Hayal Alotaibi

**Affiliations:** 1 Department of Chemistry, College of Science, Al-Nahrain University, Baghdad 64021, Iraq; alaaalqaycy7@gmail.com; 2 Cornea Research Chair, Department of Optometry, College of Applied Medical Sciences, King Saud University, P.O. Box 10219, Riyadh 11433, Saudi Arabia; 3 Polymer Research Unit, College of Science, Al-Mustansiriyah University, Baghdad 10052, Iraq; drahmed625@gmail.com; 4 Department of Medical Instrumentation Engineering, Al-Mansour University College, Baghdad 64021, Iraq; dinasaadi86@gmail.com; 5 National Center for Petrochemicals Technology, King Abdulaziz City for Science and Technology, P.O. Box 6086, Riyadh 11442, Saudi Arabia

**Keywords:** tin compounds, valsartan, poly(vinyl chloride), additives, average molecular weight, weight loss, functional group index

## Abstract

Poly(vinyl chloride) is a common plastic that is widely used in many industrial applications. Poly(vinyl chloride) is mixed with additives to improve its mechanical and physical properties and to enable its use in harsh environments. Herein, to protect poly(vinyl chloride) films against photoirradiation with ultraviolet light, a number of tin complexes containing valsartan were synthesized and their chemical structures were established. Fourier-transform infrared spectroscopy, weight loss, and molecular weight determination showed that the non-desirable changes were lower in the films containing the tin complexes than for the blank polymeric films. Analysis of the surface morphology of the irradiated polymeric materials showed that the films containing additives were less rough than the irradiated blank film. The tin complexes protected the poly(vinyl chloride) films against irradiation, where the complexes with high aromaticity were particularly effective. The additives act as primary and secondary stabilizers that absorb the incident radiation and slowly remit it to the polymeric chain as heat energy over time at a harmless level.

## 1. Introduction

Plastics are extensively used as replacements for metals, glass, and wood in many modern applications [1]. Plastics have unique performance and superior properties compared with other materials [2]. The properties of plastic such as the toughness, rigidity, density, color, and transparency can be controlled during the manufacture process. Moreover, plastics can be cheaply produced and last for a long time. The most common plastics are polyethylene, polyethylene terephthalate, polypropylene, polystyrene, and poly(vinyl chloride) (PVC) [3]. PVC has a high chlorine content (ca. 57% by weight) and is thus non-combustible [1]. Therefore, PVC can be used in furniture; construction; and many construction applications such as upholstery, pipes, windows shutters, roofing foils, flooring, and fire retardants [4]. In addition, PVC has good mechanical and chemical properties, is easy to produce in large quantitates, and resists ecological strain cracking [5].

The performance of PVC can be enhanced by incorporating additives to enable its use in outdoor applications. PVC can be mixed with plasticisers to enable the production of flexible polymeric materials for certain applications [4,5]. PVC undergoes gradual degradation in harsh environments, such as under exposure to heat and direct ultraviolet (UV) light for a long period [6]. The degradation of PVC leads to a reduction in its mechanical integrity, change in color, and formation of micro-cracks within the surface. Therefore, PVC cannot be used on its own and must be combined with stabilizers to enhance its photostability [7]. The additives should be easy and cheap to produce and well incorporated within the PVC polymeric chains. Further, the additives should not alter the color of PVC and should be non-volatile, chemically stable, non-toxic, and should not pollute the surrounding environment. The most common PVC industrial additives act as smoke suppressors, flame retardants, thermal and impact modifiers, heat stabilizers, UV stabilizers, screeners, absorbers, and free radical scavengers [8,9,10]. PVC stabilizers are classified into various types [8], that is, primary stabilizers that deactivate the allylic chlorides that are generated in the photodegradation of the polymeric chains and secondary additives that act as scavengers of chloride radicals and hydrogen chloride [11]. Many non-toxic organic materials have been used as PVC additives [12].

**Figure 1:**
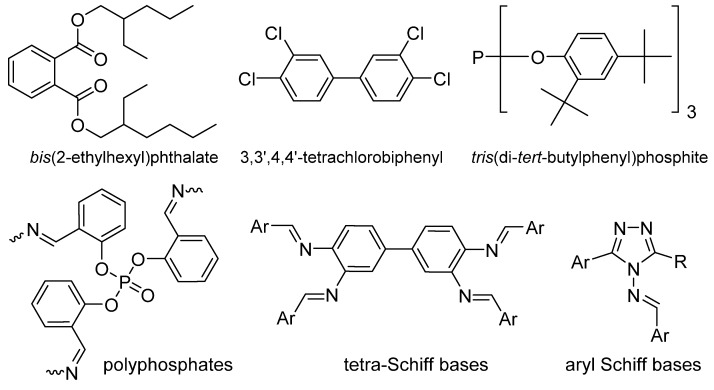
Some common poly(vinyl chloride) (PVC) additives.

*bis*(2-Ethylhexyl)phthalate (Figure 1) can be obtained from phthalic acid and has been used as a PVC plasticizer in the past. It is non-volatile, oily, has a low production cost, and is compatible with PVC [8]. However, the safety and health hazards associated with phthalates hinder their use in medicinal products (e.g. blood bags). Tetrachlorobiphenyl (Figure 1) has been used in the past as a PVC flame retardant and stabilizer [13]. However, chlorinated aromatics were banned owing to their carcinogenic and environmentally hazardous nature [13]. Stabilizers containing metals are common. For example, stabilizers containing a mixture of barium and zinc are considered non-hazardous under normal use. However, a co-stabilizer is required along with the barium and zinc mixture, for example [14,15,16]. Alternative stabilizers have been used to replace those that pose a risk to the environment and humans. For example, *tris*(di-*tert*-butylphenyl)phosphite (Figure 1) has been used as a PVC additive and antioxidant on the commercial scale [17].

Recently, various additives (Figure 1), including polyphosphates [18,19,20], Schiff bases [21,22,23,24,25], aromatic compounds [26,27], and organic–metal complexes [28,29,30,31], were investigated for use as PVC stabilizers. Some success has been achieved; however, research into the design of new and efficient PVC stabilizers for use on the commercial scale is ongoing. In the current work, we report the synthesis of a number of new tin complexes containing valsartan. The substituents on the synthesized tin complexes are varied to include aliphatic (butyl groups) or aromatic substituents (phenyl groups).

Valsartan is commercially available, non-toxic, highly aromatic, and contains a high level of oxygen and nitrogen (heteroatoms). Therefore, it was expected that the synthesized tin complexes, and in particular those with a high degree of aromaticity, would act as efficient PVC stabilizers.

## 2. Materials and Methods

### 2.1. General

Chemicals, reagents, and solvents were obtained from Merck (Gillingham, UK). PVC (${\bar{M}}_{V}$ = *ca*. 171,000) was supplied by Petkim Petrokimya (Istanbul, Turkey). An AA-6880 Shimadzu atomic absorption flame spectrophotometer (Shimadzu, Tokyo, Japan) was used to measure the tin content of the complexes. The FTIR spectra were recorded on an FTIR 8300 Shimadzu spectrophotometer (Shimadzu, Tokyo, Japan). ^1^H NMR (500 MHz) spectra were recorded on a Varian INOVA spectrometer (Palo Alto, CA, USA). ^119^Sn NMR (107 MHz) spectra were recorded on a Bruker DRX spectrophotometer (Bruker, Zürich, Switzerland). An accelerated weather-meter QUV tester obtained from Q-Panel Company (Homestead, FL, USA) was used to irradiate the PVC samples with UV light (λ_max_ = 365 nm) at 25 °C. An Ostwald U-Tube Viscometer was used to measure the viscosity of PVC. The surface morphology of the PVC films was inspected using a Veeco system (Plainview, NY, USA) and a TESCAN MIRA3 LMU instrument (Kohoutovice, Czech Republic) at an accelerating voltage of 10 kV.

### 2.2. Synthesis of Tin Complexes **1** and **2**

**Figure 2:**
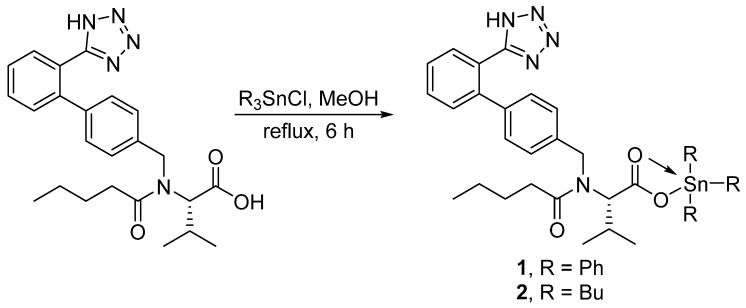
Synthesis of tin complexes **1** and **2**.

A mixture of valsartan (0.87 g; 2.0 mmol) and triphenyltin or tributyltin chloride (2.0 mmol) in boiling methanol (MeOH; 30 mL) was stirred for 6 h. The solid obtained after cooling the mixture to 25 °C was filtered, washed with MeOH, and dried to give **1** or **2** (Figure 2).

### 2.3. Synthesis of Tin Complexes **3** and **4**

**Figure 3:**
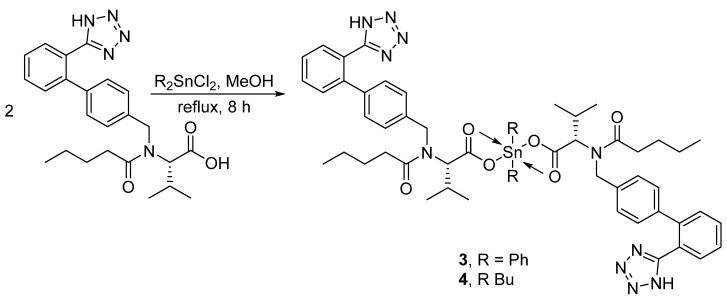
Synthesis of tin complexes **3** and **4**.

A mixture of valsartan (0.87 g; 2.0 mmol) and diphenyltin or dibutyltin chloride (1.0 mmol) in boiling MeOH (30 mL) was stirred for 8 h. The solid obtained was filtered, washed with MeOH, and dried to give **3** or **4** (Figure 3).

### 2.4. Preparation of PVC Films

A mixture of PVC (5.0 g) and the tin complex (25 mg) in tetrahydrofuran (THF; 100 mL) was stirred at 25 °C for 2 h. The mixture was transferred onto glass plates with a thickness of 40 µm. The films produced were left to dry under vacuum for 18 h.

### 2.5. Assessment of PVC Photodegradation Using FTIR Spectrophotometry

Photodegradation of the PVC films was investigated using FTIR spectrophotometry. The changes in the intensities of the absorption peak of the carbonyl (C=O; 1722 cm^−1^) and polyene (C=C; 1602 cm^−1^) groups were monitored. These peaks arise owing to the formation of small fragments containing C=O and C=C groups generated during PVC photooxidation. The change in the intensity of these peaks was monitored relative to the intensity of a standard peak (the C–H bond of the CH_2_ groups; 1328 cm^−1^). Equation (1) was used to calculate the functional group (C=O or C=C) index (*I_s_*) from the absorbance of the functional group (*A*_s_) and that for the standard peak (*A*_r_) [32].

(1) $$I_{s}=A_{s}/A_{r}$$

### 2.6. Assessment of PVC Photodegradation Using Weight Loss

The weight loss of the PVC films due to photodegradation was calculated from the weight of PVC before (*W_0_*) and after irradiation (*W_t_*) using Equation (2) [30].

(2) $$Weight\;loss\;\left(\%\right)=\left[\left(W_{0}-W_{t}\right)/W_{0}\right]\times 100$$

### 2.7. Assessment of PVC Photodegradation Using Average Molecular Weight (${\bar{M}}_{V})$

The ${\bar{M}}_{V}$ of PVC after irradiation was calculated from the intrinsic viscosity [*η*] of the solution of the polymeric materials using Equation (3), known as the Mark–Houwink equation [33].

(3) $$\left[\eta \right]=1.63\times 10^{-2}\;M_{v}^{0.766}$$

## 3. Results and Discussion

### 3.1. Synthesis of Tin Complexes **1**–**4**

The reaction of valsartan and the appropriate tributyltin or triphenyltin chloride (in a 1:1 ratio) in boiling MeOH for 6 h gave **1** or **2** (Figure 2) in 82% and 79% yield, respectively (Table 1). Similarly, reaction of excess valsartan (two mole equivalents) and the appropriate dibutyltin or diphenyltin chloride for 8 h in refluxing MeOH gave **3** and **4** (Figure 3) in 75% and 88% yield, respectively (Table 1). The purity and elemental composition of the synthesized tin complexes **1**–**4** were confirmed by elemental analysis. The color, melting point, yield, and elemental analysis data for the tin complexes **1**–**4** are summarized in Table 1.

The FTIR spectra of **1**–**4** (Figures S1–S4) show absorption bands corresponding to the symmetrical and asymmetrical vibrations of the carbonyl group in the regions of 146–1477 and 1732–1735 cm^−1^, respectively (Table 2) [34]. For valsartan, these absorption bands appeared at lower wavenumbers (1442 and 1670 cm^−1^, respectively), which clearly indicated the formation of a bond between the tin atom and oxygen of the carboxylate group [35]. Indeed, the absorption bands that appeared at 443–455 cm^−1^ are attributed to the Sn–O bonds. In addition, the Sn–C absorption bands appeared in the 559–563 cm^−1^ region. The difference [*∆v* (asym – sym)] between the symmetric (sym) and asymmetric (asym) vibrational frequencies for the carbonyl group was in the range of 258–270 cm^−1^. The value of *∆v* indicates that valsartan acts as an asymmetric bi-dentate ligand [36].

The ^1^H NMR spectra of **1**–**4** (Figures S5–S8) show the absence of the proton resonance at 12.63 ppm for the carboxylic group of valsartan [37]. Clearly, the tin complexes were produced as a result of replacement of the carboxylic group proton with a tin atom. The ^1^H NMR spectra of **1**–**4** showed the presence of protons of both valsartan and the substituents (phenyl and butyl moieties) at the expected chemical shifts (Table 3).

The ^119^Sn NMR spectra of **1**–**4** (Figures S9–S12) showed characteristic signals at −137.8 and −131.2 ppm owing to the tin atom in **1** and **2**, respectively. These chemical shifts (Table 3) revealed the formation of five-coordinated complexes [38,39,40]. For **3** and **4**, the signals corresponding the Sn atom appeared at −406.1 and −218.1 ppm, respectively. The chemical shifts for these signals indicated the formation of six coordinated complexes. Clearly, the geometry of the complexes affects the chemical shifts as a result of the shielding effect of the tin atom and substituents [38,39,40].

### 3.2. Assessment of Photodegradation of PVC Using Energy Dispersive X-ray (EDX) Mapping

The elemental composition of **1**–**4** was analyzed using energy dispersive X-ray (EDX). EDX confirmed the elements within complexes **1**–**4** (Figures S13–S18) [41]. PVC was mixed with complexes **1**–**4** (0.5 wt.%) and thin (40 μm) films were made. The films were irradiated with UV light and EDX was used to determine the elemental composition of the polymer blends. The EDX mapping images revealed that the tin complexes were well-distributed throughout the films [42]. For the unmodified (blank) PVC, the percentage of chlorine in the films was reduced from 64.8% before irradiation to 55.8% after irradiation (300 h). These results indicate significant dehydrochlorination, where hydrogen chloride was eliminated from the blank PVC as a result of photodegradation. After irradiation, the reduction in the chlorine content of the PVC films containing complexes **1**–**4** was lower compared with that of the blank PVC film. The chlorine content was highest (56.4%) in the case of the irradiated PVC/**1** blend. Complex **1**, which is highly aromatic (three phenyl, two aryl, and one tetrazole moieties) was the most efficient additive for stabilizing the polymeric materials. It has been reported that additives containing aromatic moieties are more efficient PVC photostabilizers compared with the corresponding ones containing aliphatic residues [31]. Complex **1** absorbs UV irradiation directly and releases the adsorbed energy over a long period of time at a rate that is not harmful to the PVC chains.

### 3.3. Assessment of Photodegradation of PVC Using FTIR Spectrophotometry

**Figure 4:**
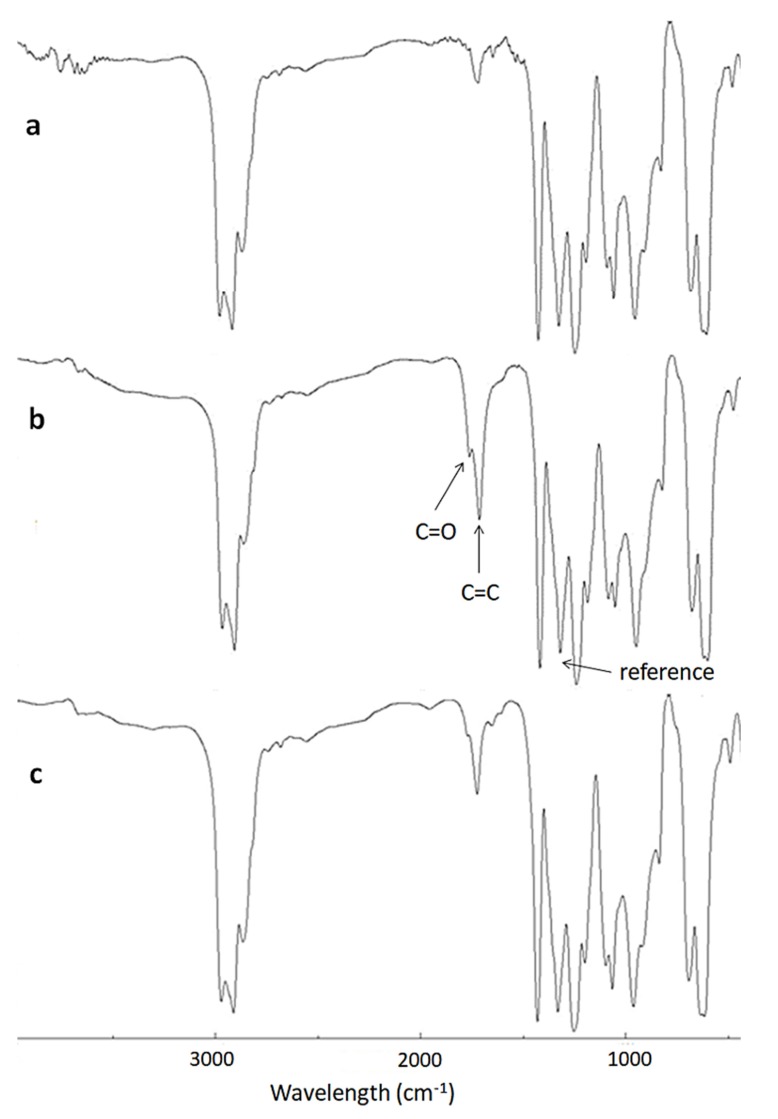
FTIR spectra of (**a**) poly(vinyl chloride) (PVC) film before irradiation, (**b**) PVC film after irradiation (300 h), and (**c**) PVC + **1** blend after irradiation (300 h).

PVC undergoes photooxidative degradation upon irradiation in the presence of an oxygen source [43,44]. This process leads to the formation of small polymeric fragments containing carbonyl (C=O; carboxyl and ketone) and polyene (C=C; carbon–carbon double bond residues) groups [43,44]. Such functional groups can be detected using FTIR spectroscopy. In addition, the intensity of the FTIR signals can be monitored during the photooxidation of PVC and compared with the intensity of the signals of the C–H bond of the CH_2_ moieties (1328 cm^−1^) within the polymeric chains. The absorption of the C–H bond is not altered during the irradiation process. Figure 4 shows that the intensity of the signals of both the C=O (1722 cm^−1^) and C=C (1602 cm^−1^) groups was significantly higher for the irradiated blank PVC film. For the PVC film containing complex **1**, it was clear that the intensity of the peaks of both functional groups was significantly lower than that of the corresponding peaks that appeared for the irradiated PVC (blank) film.

**Figure 5:**
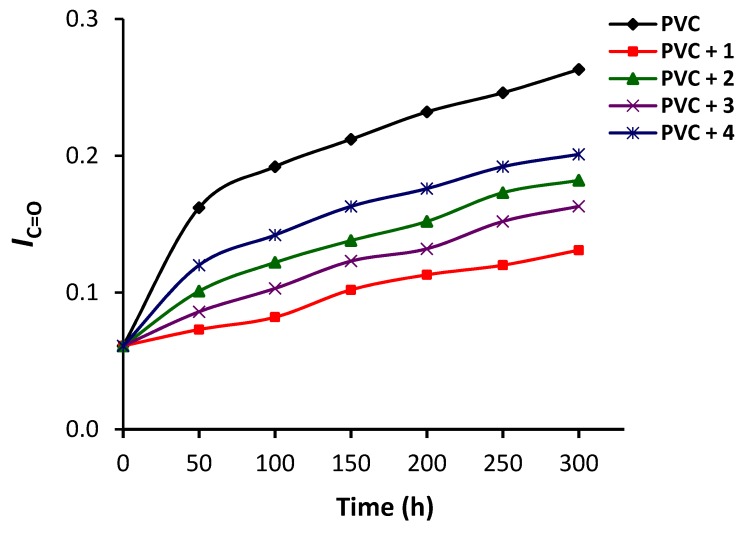
Changes in the *I*_C=O_ index for PVC films.

**Figure 6:**
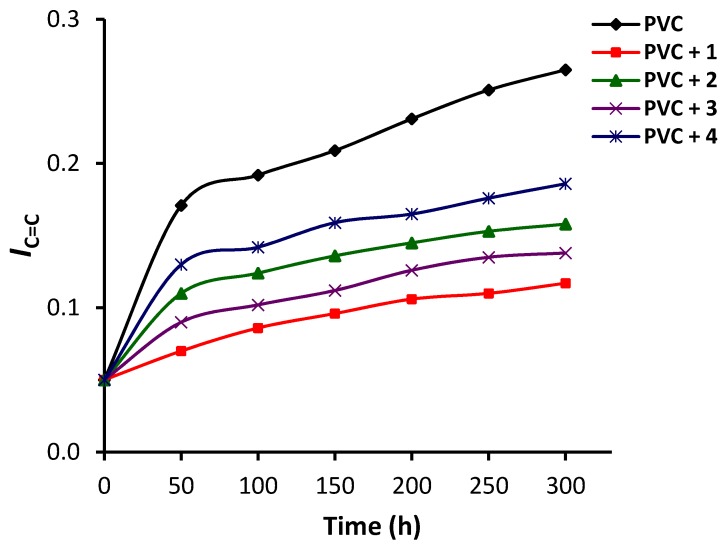
Changes in the *I*_C=C_ index for PVC films.

Equation (1) was used to calculate the functional group indices (*I*_C=O_ and *I*_C=C_) for the blank PVC and those containing complexes **1**–**4** at 50 h intervals for an irradiation period of up to 300 h. The *I_C=O_* and *I*_C=O_ values for the PVC films were plotted against the irradiation time at 50 h intervals (Figure 5 and Figure 6). Both *I*_C=O_ and *I*_C=O_ changed significantly for the PVC film in the absence of any additives compared with the PVC films containing **1**–**4** as additives. Clearly, complexes **1**–**4** stabilized PVC substantially. For example, the *I*_C=O_ after 300 h of irradiation was 0.26, 0.13, 0.18, 0.16, and 0.20 for the PVC, PVC/**1**, PVC/**2**, PVC/**3**, and PVC/**4** films, respectively. Similarly, the *I*_C=C_ for blank PVC was 0.27 after irradiation (300 h) compared with 0.12, 0.16, 0.14, and 0.19 for the PVC/**1**, PVC/**2**, PVC/**3**, and PVC/**4** films, respectively. Clearly, the minimum change in the indices of the C=O and C=C groups was achieved when complex **1** (highly aromatic) was used. The efficiency of complexes **1**–**4** for photostabilizing PVC against irradiation followed the order: **1** (triphenyltin) > **3** (diphenyltin) > **2** (tributyltin) > **4** (dibutyltin).

### 3.4. Assessment of Photodegradation of PVC Using Weight Loss

**Figure 7:**
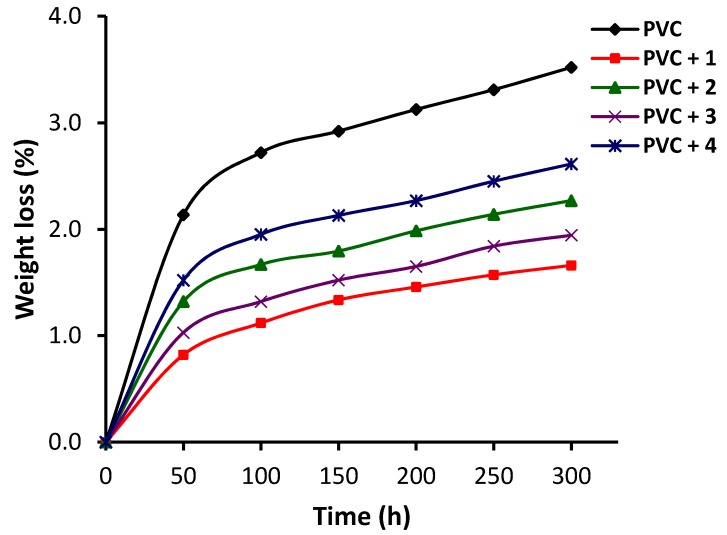
Changes in weight loss of PVC films.

The photooxidation of PVC causes cross-linking of the polymeric chains owing to the production of free radical moieties. As a result, hydrogen chloride (dehydrochlorination) and volatile small organic residues are eliminated, accompanied by PVC discoloration. Such processes lead to weight loss at a variety of relatively high temperatures [45,46,47]. To determine the efficiency of complexes **1**–**4** as stabilizers, the PVC films were irradiated and the weight loss was calculated at 50 h intervals during irradiation using Equation (2). The results obtained are presented in Figure 7. The PVC weight loss was sharp at the beginning of irradiation (50 h) and increased gradually and reached a maximum after 300 h of continuous irradiation. The PVC weight loss was highest (3.5%) for unmodified PVC. In the presence of complexes **1**–**4**, the PVC weight loss ranged from 1.7%−2.6% after 300 h of continuous irradiation. The PVC weight loss was lowest (1.7%) when complex **1** was used as the additive. The weight loss percentage for the PVC/**2**, PVC/**3**, and PVC/**4** blends was 2.3%, 1.9%, and 2.6%, respectively. Clearly, the complexes, and in particular the highly aromatic additives (complex **1** and **3**), enhanced the photostability of the PVC films significantly.

### 3.5. Assessment of PVC Photodegradation Using Viscosity Average Molecular Weight (${\bar{M}}_{V}$)

**Figure 8:**
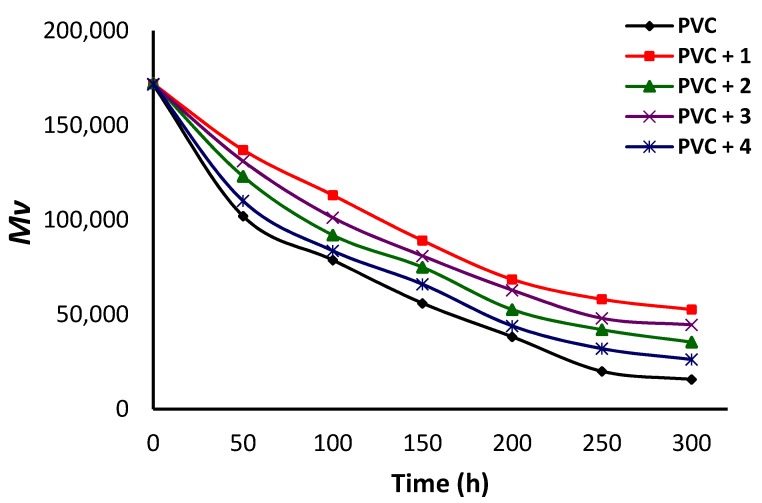
Changes in the ${\bar{M}}_{V}$ for PVC films.

Photodegradation of PVC leads to a decrease in its molecular weight as a result of chain scission [48]. The viscosity of PVC in solution is used as a measure of the ${\bar{M}}_{V}$. The PVC films irradiated for different periods were dissolved in THF and their viscosity was measured using a viscometer [33]. The ${\bar{M}}_{V}$ for each film at different irradiation times (from 50 to 300 h) was calculated using Equation (3). The results are presented in Figure 8.

A clear decrease in the ${\bar{M}}_{V}$ was observed during the irradiation process and was more pronounced for the blank PVC film. For example, the ${\bar{M}}_{V}$ for blank PVC was approximately 171,300 at the start of the irradiation process and declined to 78,800 after 100 h, to 38,300 after 200 h, and to only 15,700 at the end of the irradiation process (300 h). At the end of the irradiation process, the ${\bar{M}}_{V}$ for the PVC/**1**, PVC/**2**, PVC/**3**, and PVC/**4** blends was 52,600, 35,400, 44,600, and 26,300, respectively. The ${\bar{M}}_{V}$ for the blank PVC film decreased by more 90% after the irradiation process, compared with 70% when complex **1** was used as the additive. Clearly, the tin complexes, and in particular **1**, stabilized PVC against irradiation to a significant degree.

### 3.6. Surface Analysis of PVC

**Figure 9:**
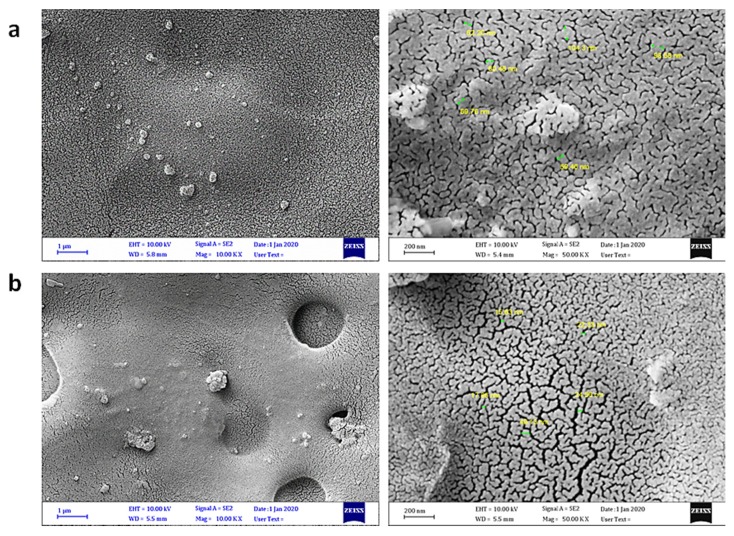
Field-emission scanning electron microscopy (FESEM) images of (**a**) PVC film before irradiation and (**b**) PVC film after irradiation.

**Figure 10:**
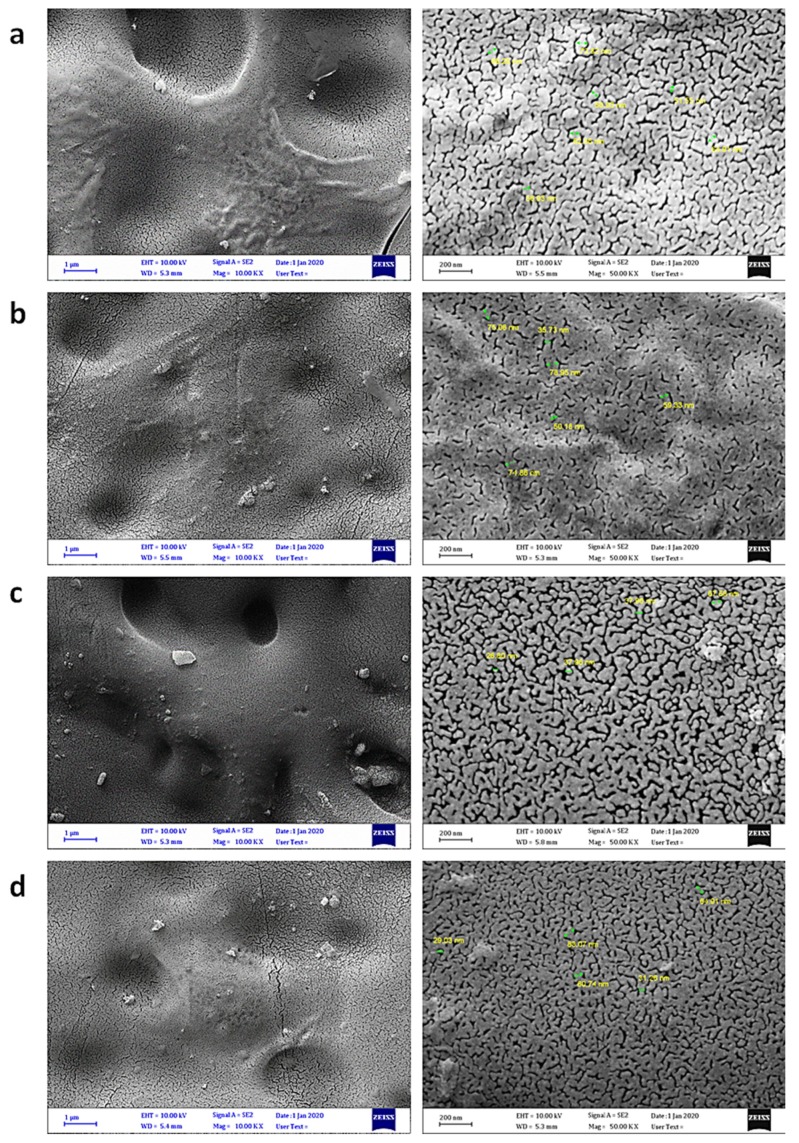
FESEM images of (**a**) PVC + **1**, (**b**) PVC + **2**, (**c**) PVC + **3**, and (**d**) PVC + **4** films after irradiation.

Scanning electron microscopy (SEM) can be used to provide less distorted, clear, and high-resolution images of the particles in materials, and is a useful technique for investigating the variation of the surface, particle size and shape, homogeneity, and cross sections within the blends [49,50,51,52]. The surface morphology of the PVC films was examined by field-emission scanning electron microscopy (FESEM). Figure 9 shows the FESEM images of the surface of the PVC film before and after irradiation. It has been reported that non-irradiated polymers have a smooth surface and a high level of homogeneity [19]. Indeed, the surface of the blank PVC film before irradiation was smoother, flatter, less lumpy, and more homogeneous compared with that obtained after irradiation. The roughness and cracks formed on the PVC surface after irradiation are the result of bond breaking within the polymeric chains and elimination of hydrogen chloride [53]. Figure 10 shows the FESEM images of the particles within the PVC films containing complexes **1**–**4** after irradiation, at two magnification powers. The surface of the PVC/**1** blend was more or less smooth and clean, similar to that for the non-irradiated PVC film. Clearly, complexes **1**–**4** were very effective in stabilizing PVC against irradiation. The other complexes provided some degree of stabilization to the PVC films.

**Figure 11:**
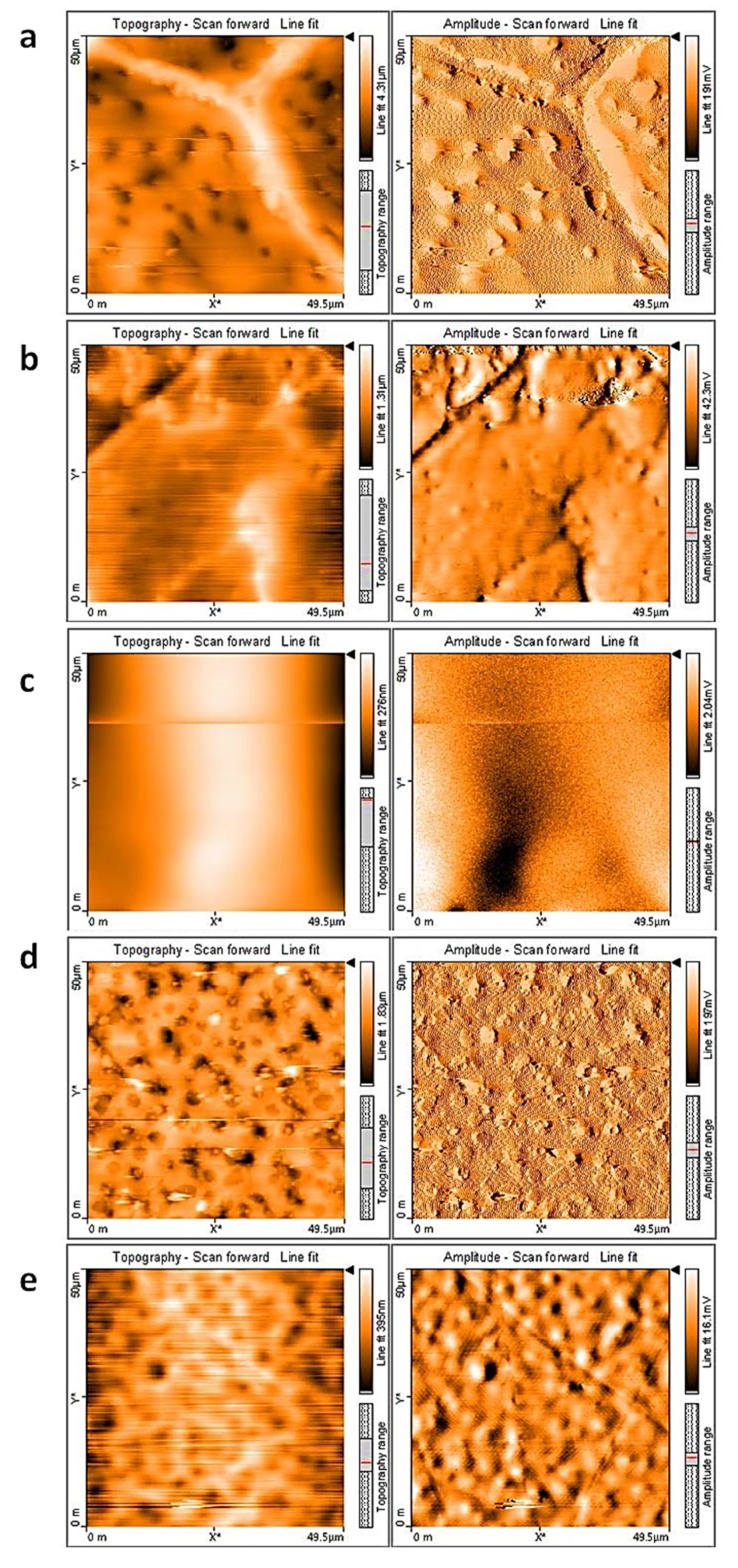
Atomic force microscopy (AFM) images of (**a**) PVC, (**b**) PVC + **1**, (**c**) PVC + **2**, (**d**) PVC + **3**, and (**e**) PVC + **4** films.

The surface morphology of the PVC film was also observed using atomic force microscopy (AFM). AFM provides useful information about the features and roughness of the PVC surface. Irradiation of PVC for a long duration leads to bond breaking to produce a rough and broken surface [54,55]. Figure 11 shows the topographic AFM images (two- and three-dimensional) of the surface of the PVC films after 300 h of irradiation. The addition of complexes **1**–**4** clearly improved the photostability of the PVC films. The surface of the irradiated PVC blends containing complexes **1**–**4** was less rough than that of the blank PVC film. The roughness factor (*R*_q_) for the PVC, PVC/**1**, PVC/**2**, PVC/**3**, and PVC/**4** films was 452, 61, 95, 80, and 112, respectively. Clearly, the use of complex **1** improved the roughness of the PVC film 7.4-fold. This improvement in *R*_q_ is better than that reported with many PVC additives (Table 4). Clearly, complexes **1**–**4** are better PVC photostabilizers compared with triazole-3-thiol Schiff bases [21], biphenyl tetraamine Schiff bases [22], naproxen tin complexes [30], melamine Schiff bases [24], 2-(4-isobutylphenyl)propanoate tin complexes [56], and furosemide tin complexes [28].

### 3.7. PVC Photostabilization Mechanisms

**Figure 12:**
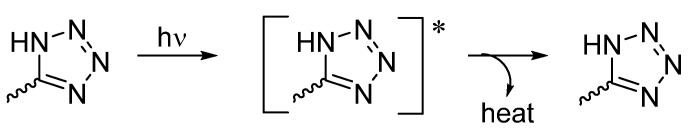
Function of tetrazole unit as a UV absorber.

The use of tin complexes **1**–**4** as additives significantly reduced photodegradation of the PVC films. The effectiveness of the synthesized tin complexes as PVC photostabilizers followed the order: **1** > **3** > **2** > **4**. Complexes **1** and **3** are highly aromatic (phenyl moieties), while complexes **2** and **4** contain butyl substituents (aliphatic moieties). In addition, complexes **1**–**4** contain two aryl and one tetrazole ring within their skeletons. Therefore, these complexes are able to absorb UV light directly. After irradiation, the complexes re-emitted the absorbed radiation as a heat energy over a period of time at a rate that is not harmful to the PVC polymeric chains (Figure 12). The high-energy state of the tetrazole, aryl, or phenyl ring within complexes **1**–**4** can be stabilized by the resonance of the aromatic moieties [26].

**Figure 13:**
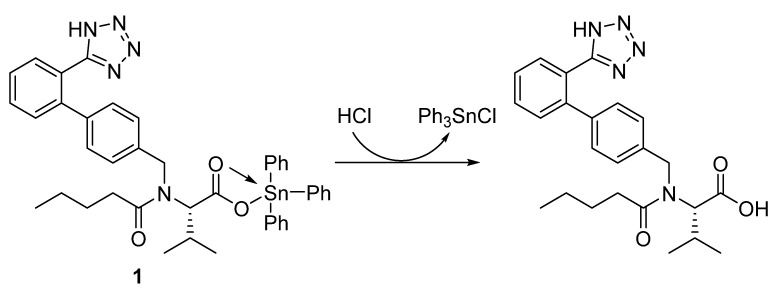
Function of complex **1** as a hydrogen chloride scavenger.

Complexes **1**–**4** contain the tin atom, which acts as an acidic center. Tin abstracts the chloride ion from hydrogen chloride that is eliminated from the PVC chains upon irradiation to produce a stable substituted tin chloride (Figure 13; for triphenyltin complex **1**). Therefore, the tin complexes, and **1** in particular, are secondary PVC photostabilizers and act as hydrogen chloride scavengers [7,28].

**Figure 14:**
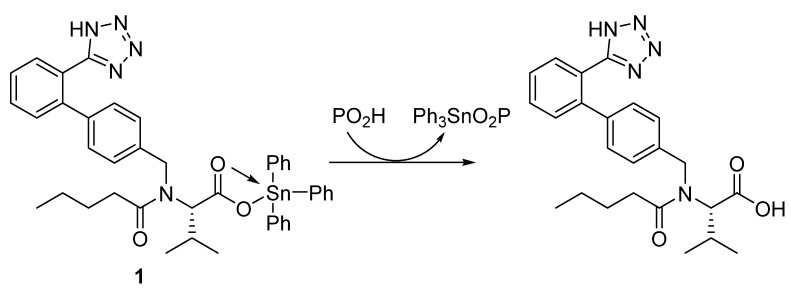
Function of complex **1** to induce hydroperoxide decomposition.

PVC undergoes photooxidation in the presence of oxygenated species such as hydrogen peroxides (PO_2_H) [55,56]. The tin complexes can induce the decomposition of hydroperoxides by displacing the acidic tin atom within the additive (Figure 14; for triphenyltin complex **1**). This process inhibits PVC photodegradation significantly.

**Figure 15:**
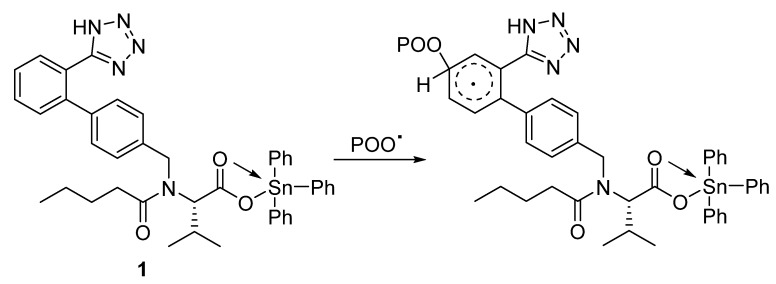
Function of complex **1** as a radical scavenger.

Peroxide radicals (POO^•^) have a negative impact on the PVC films and lead to the formation of various photooxidative products. The synthesized tin complexes can act as radical scavengers. The intermediate containing both the peroxide radical (chromophore) and the aryl moieties within the additives (Figure 15; for triphenyltin complex **1**) is highly stable via resonance [57]. Therefore, complexes **1**–**4** inhibit PVC photooxidation and provide a degree of stabilization against irradiation.

**Figure 16:**
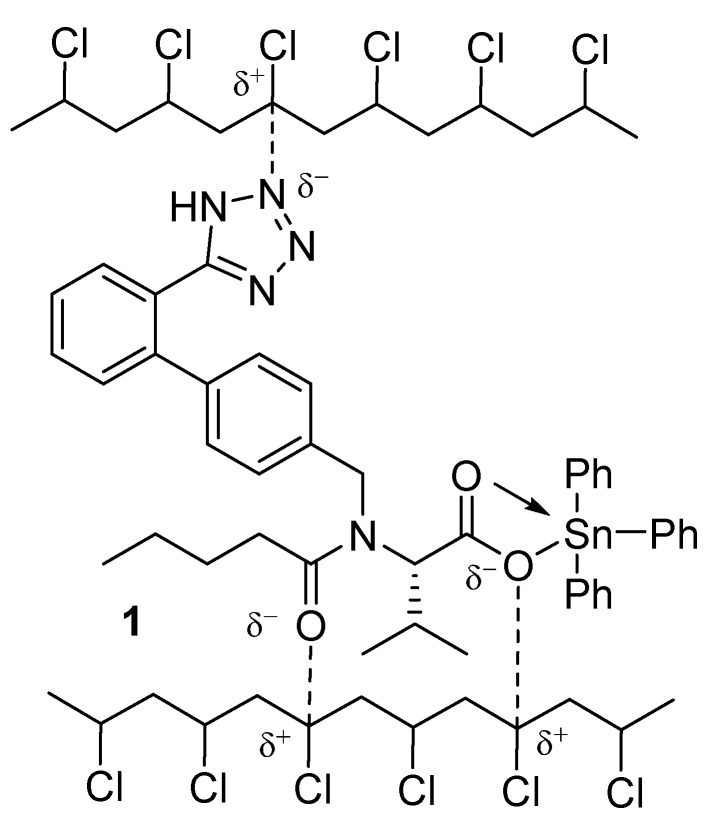
Interaction between complex **1** and PVC.

The polarity of the C–Cl bonds within the PVC chains and that of the nitrogen atoms in the tetrazole ring and the oxygen of carboxylate and amide groups might facilitate attractive interactions between PVC and the additives (Figure 16; for triphenyltin complex **1**). This attraction may augment energy transfer from the polymeric chains to the additives before the energy from photoirradiation can be dissipated [28]. However, this hypothesis does not take into account the complications due to steric hindrance within macromolecules.

## 4. Conclusions

A number of tin complexes containing valsartan were synthesized in high yields using simple procedures. The synthesized tin complexes varied in the number and type of substituents. The chemical structures and elemental compositions of the tin complexes were established using various analytical tools. The effectiveness of the new tin complexes as photostabilizers for poly(vinyl chloride) films was tested after irradiation with ultraviolet light for up to 300 h. The undesirable changes in the carbonyl and polyene functional group indices, weight, and molecular weight of the polymeric films were significantly reduced in the presence of the tin complexes. The surface of the polymeric blends containing the tin complexes was smoother, flatter, and less rough than that of the blank film. Clearly, the synthesized tin complexes, and in particular those with a high level of aromaticity (phenyl derivatives), act as photostabilizers to protect the films against photodegradation. The main function of the tin complexes is to directly absorb ultraviolet irradiation, with subsequent slow release of the energy over time in a manner that does not degrade the polymeric chains and free radicals and hydrogen chloride scavengers. Valsartan tin complexes could be used as poly(vinyl chloride) photostabilizers in an industrial scale, but the production cost needs to be assessed.

## Figures and Tables

**Table 1:** Color, melting point, yield, and elemental analysis of **1**–**4**.

Complex	Color	Melting Point (°C)	Yield (%)	Found (Calculated) (%)			
				C	H	N	Sn
**1**	white	257–259	82	64.29 (64.30)	5.50 (5.52)	8.92 (8.93)	15.10 (15.13)
**2**	white	112–114	79	59.65 (59.68)	7.62 (7.65)	9.64 (9.67)	16.34 (16.38)
**3**	off- white	103–105	75	63.07 (63.11)	5.79 (5.83)	12.23 (12.27)	10.37 (10.40)
**4**	off- white	76–78	88	61.01 (61.04)	6.74 (6.77)	12.68 (12.71)	10.73 (10.77)

**Table 2:** Select FTIR spectral data for **1–4**.

Complex	Wavenumber (cm^−1^)				
	C=O sym	C=O asym	*∆v* (asym − sym)	Sn–C	Sn–O
**1**	1477	1735	258	559	447
**2**	1462	1732	270	563	455
**3**	1473	1735	262	559	443
**4**	1477	1735	258	559	447

**Table 3:** ^1^H and ^119^Sn NMR spectral data for **1–4**.

Complex	NMR (DMSO-*d_6_*; δ in ppm and *J* in Hz)	
	^1^H (500 MHz)	^119^Sn (107 MHz)
**1**	7.73–7.06 (m, 23H, Ar), 6.95 (s, exch., 1H, NH), 4.62 (s, 2H, CH_2_), 4.47 (d, *J* = 7.2 Hz, 1H, CH), 2.22 (br, 1H, CH), 2.14 (t, *J* = 7.4 Hz, 2H, CH_2_), 1.40–1.29 (m, 4H, CH_2_CH_2_), 0.94 (d, *J* = 7.2 Hz, 6H, 2 Me), 0.78 (t, *J* = 7.4 Hz, 3H, Me)	−137.8
**2**	7.66–7.55 (m, 6H, Ar), 7.20 (d, *J* = 8.1 Hz, 1H, Ar), 7.08 (d, *J* = 8.1 Hz, 1H, Ar), 6.95 (s, exch., 1H, NH), 4.63 (s, 2H, CH_2_), 4.51 (d, *J* = 7.2 Hz, 1H, CH), 2.21 (br, 1H, CH), 2.08 (m, 2H, CH_2_), 1.60–1.30 (m, 22H, 11 CH_2_), 1.13–0.85 (m, 18H, 6 Me)	−131.2
**3**	7.70–7.53 (m, 8H, Ar), 7.37–7.00 (m, 18H, Ar), 6.97 (s, exch., 2H, 2 NH), 4.66 (s, 4H, 2 CH_2_), 4.49 (d, *J* = 7.1 Hz, 2H, 2 CH), 2.22 (br, 2H, 2 CH), 2.09 (t, *J* = 7.5 Hz, 4H, 2 CH_2_), 1.38–1.25 (m, 8H, 2 CH_2_CH_2_), 0.92 (d, *J* = 7.1 Hz, 12H, 4 Me), 0.80 (t, *J* = 7.5 Hz, 6H, 2 Me)	−406.1
**4**	7.68–7.62 (m, 12H, Ar), 7.20 (d, *J* = 8.2 Hz, 2H, Ar), 7.08 (d, *J* = 8.2 Hz, 2H, Ar), 6.97 (s, exch., 2H, 2 NH), 4.63 (s, 4H, 2 CH_2_), 4.46 (d, *J* = 7.1 Hz, 2H, 2 CH), 2.20 (br, 2H, 2 CH), 2.09 (m, 4H, 2 CH_2_), 1.65–1.29 (m, 20H, 10 CH_2_), 1.16–0.87 (m, 24H, 8 Me)	−218.1

**Table 4:** Effect of additives on roughness factor (*R*_q_; %) for poly(vinyl chloride) (PVC).

PVC Additive	Improvement in *R*_q_ (Fold)	Reference
Valsartan tin complex	7.4	[current work]
Furosemide tin complex	6.6	[28]
Ciprofloxacin tin complex	16.6	[29]
Naproxen tin complex	5.2	[30]
Telmisartan tin complex	9.4	[31]
2-(4-Isobutylphenyl)propanoate tin complex	6.2	[56]
Triazole-3-thiol Schiff base	3.3	[21]
Biphenyl tetraamine Schiff base	3.6	[22]
Melamine Schiff base	6.0	[24]
Polyphosphate	16.7	[18]

## Supplementary Materials

The following are available online at https://www.mdpi.com/2073-4360/12/4/969/s1, Figure S1: FTIR spectrum of **1**; Figure S2: FTIR spectrum of **2**; Figure S3: FTIR spectrum of **3**; Figure S4: FTIR spectrum of **4**; Figure S5: ^1^H NMR spectrum of **1**; Figure S6: ^1^H NMR spectrum of **2**; Figure S7: ^1^H NMR spectrum of **3**; Figure S8: ^1^H NMR spectrum of **4**; Figure S9: ^119^Sn NMR spectrum of **1**; Figure S10: ^119^Sn NMR spectrum of **2**; Figure S11: ^119^Sn NMR spectrum of **3**; Figure S12: ^119^Sn NMR spectrum of **4**; Figure S13: EDX graphs of blank PVC before irradiation; Figure S14: EDX graphs of PVC film after irradiation; Figure S15: EDX graphs of PVC film containing **1**; Figure S16: EDX graphs of PVC film containing **2**; Figure S17: EDX graphs of PVC film containing **3**; Figure S18: EDX graphs of PVC film containing **4**.
