# Supplementary material for: Protection of Poly(Vinyl Chloride) Films against Photodegradation Using Various Valsartan Tin Complexes
Source: Polymers (Basel). 2020 Apr 21;12(4):969. doi: 10.3390/polym12040969 (PMC7240378; doi:10.3390/polym12040969)
Supplement: Supplementary file 1 [file polymers-12-00969-s001.pdf]

polymers

MDPI

Supplementary Materials

Protection of Poly(vinyl Chloride) Films against Photodegradation using Various Valsartan Tin Complexes

Alaa Mohammed [1], Gamal A. El-Hiti [2,*], Emad Yousif [1,*], Ahmed A. Ahmed [3], Dina S. Ahmed [4] and Mohammad Hayal Alotaibi [5,*]

1 Department of Chemistry, College of Science, Al-Nahrain University, Baghdad 64021, Iraq; alaaalqaycy7@gmail.com
2 Cornea Research Chair, Department of Optometry, College of Applied Medical Sciences, King Saud University, P.O. Box 10219, Riyadh 11433, Saudi Arabia
3 Polymer Research Unit, College of Science, Al-Mustansiriyah University, Baghdad 10052, Iraq; drahmed625@gmail.com
4 Department of Medical Instrumentation Engineering, Al-Mansour University College, Baghdad 64021, Iraq; dinasaadi86@gmail.com
5 National Center for Petrochemicals Technology, King Abdulaziz City for Science and Technology, P.O. Box 6086, Riyadh 11442, Saudi Arabia
* Correspondence: gelhiti@ksu.edu.sa (G.A.E.-H.); emad_yousif@hotmail.com (E.Y.); mhhalotaibi@kacst.edu.sa (M.H.A.); Tel.: +966-11469-3778 (G.A.E.-H.); Fax: +966-11469-3536 (G.A.E.-H.)

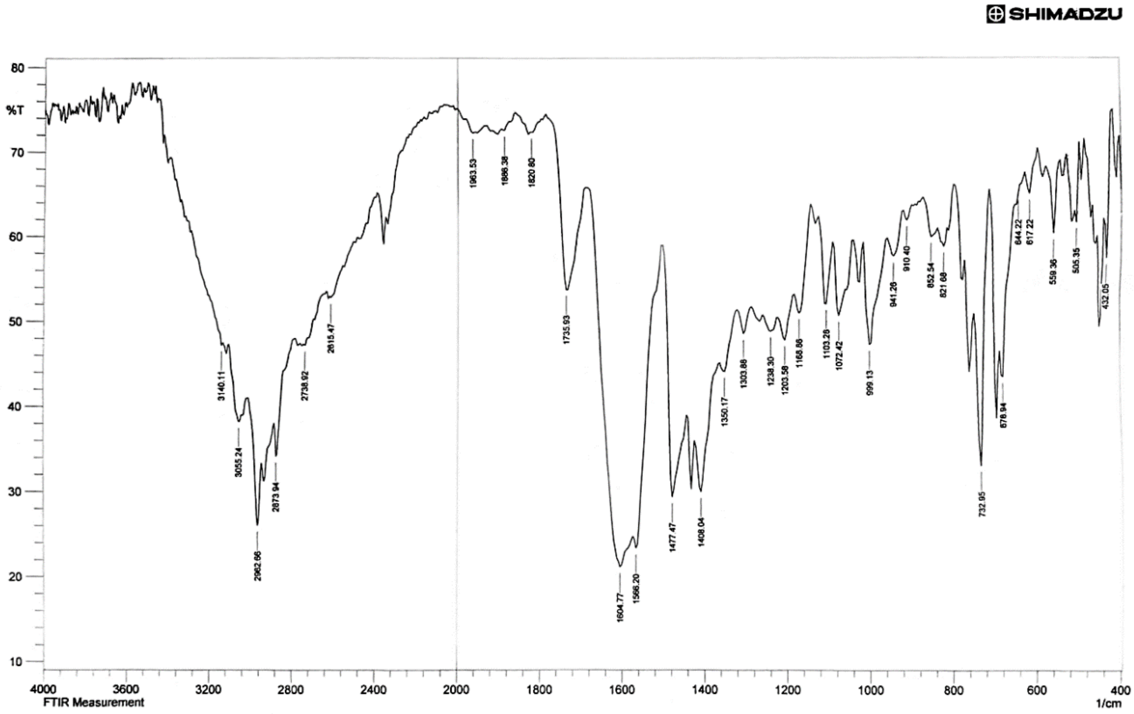

Figure S1. FTIR spectrum of **1**.

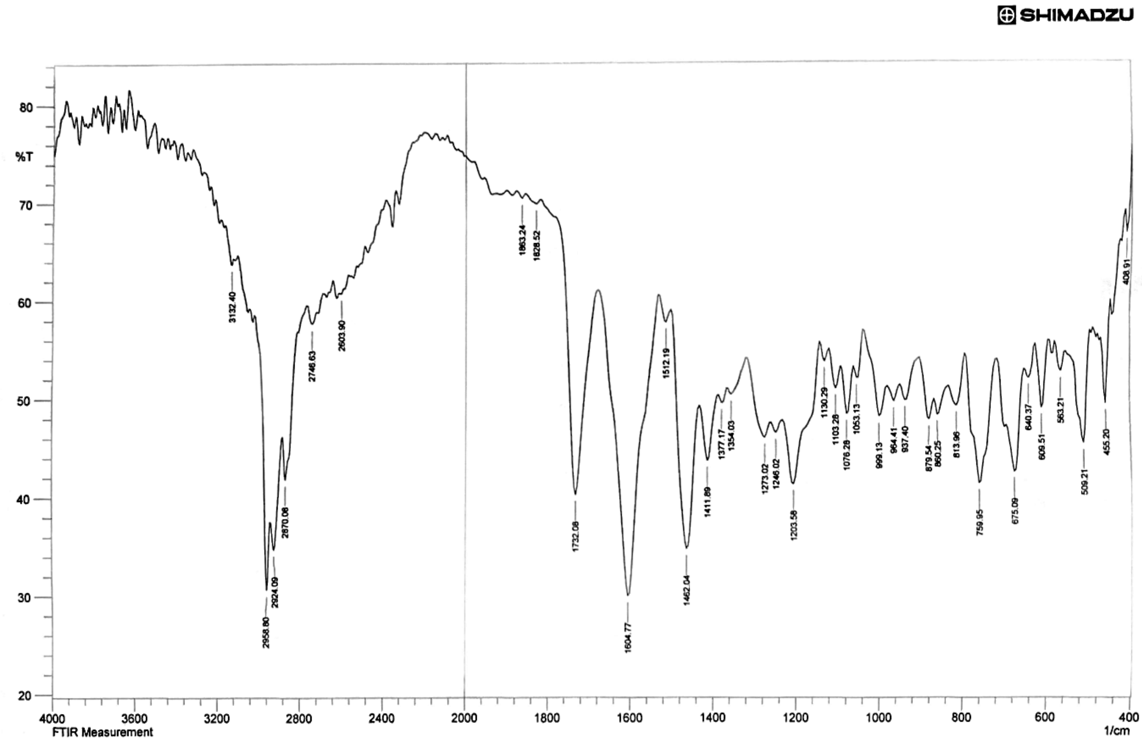

Figure S2. FTIR spectrum of **2**.

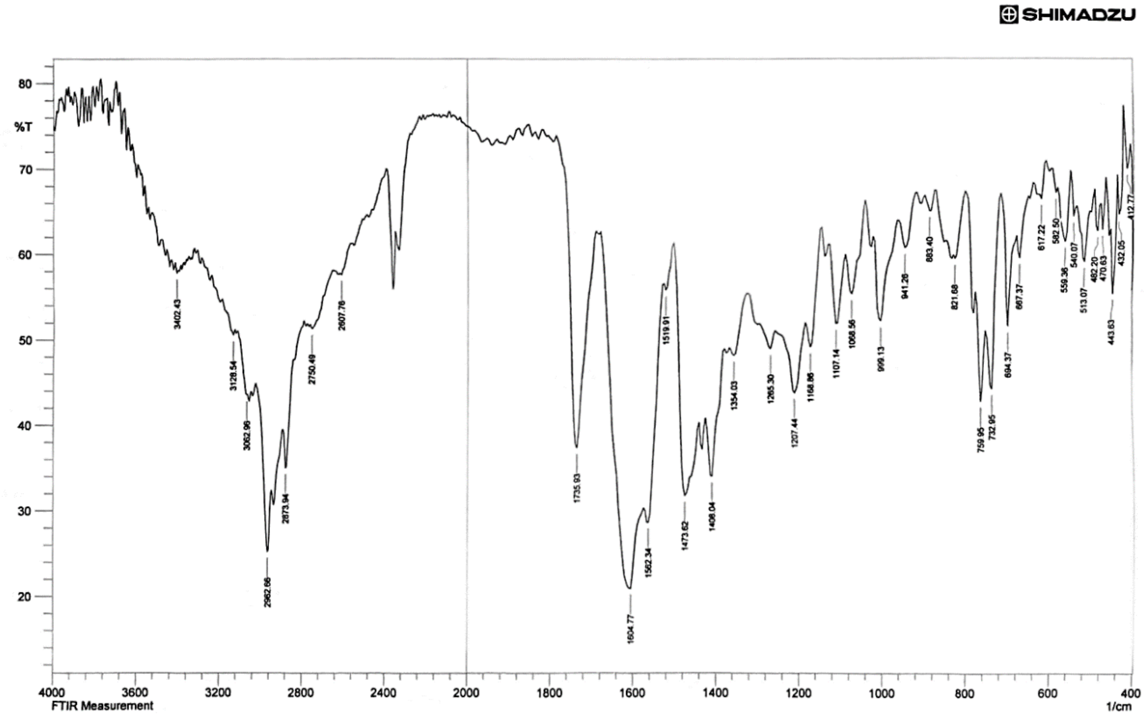

Figure S3. FTIR spectrum of **3**.

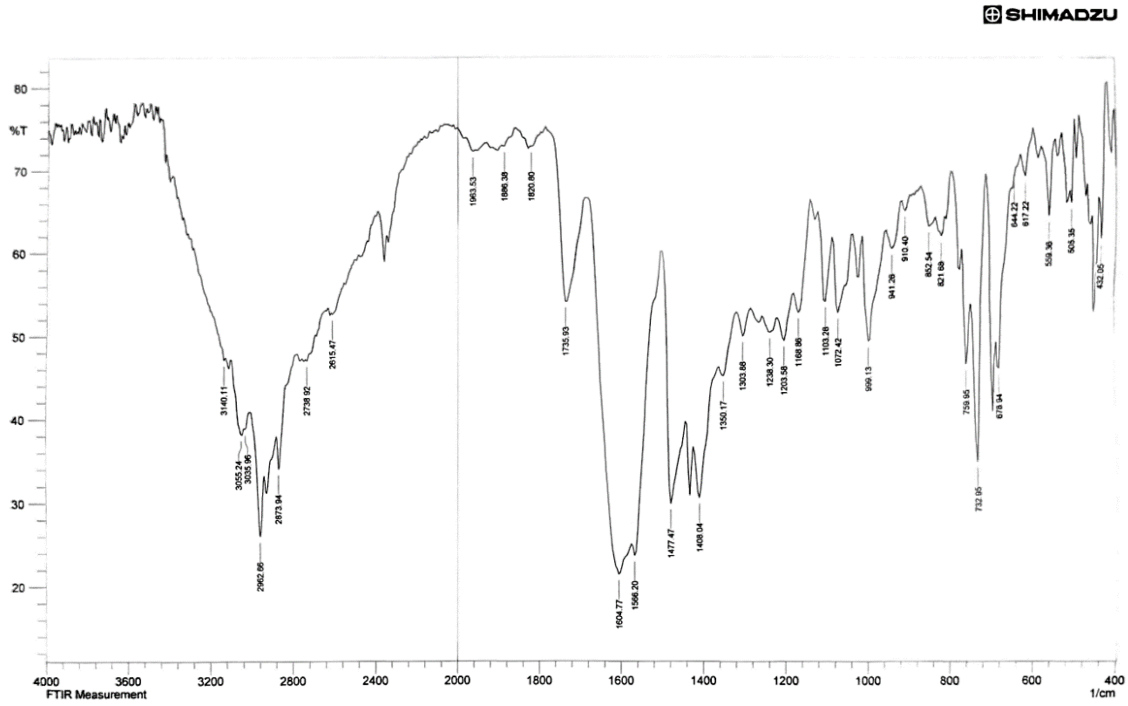

Figure S4. FTIR spectrum of **4**.

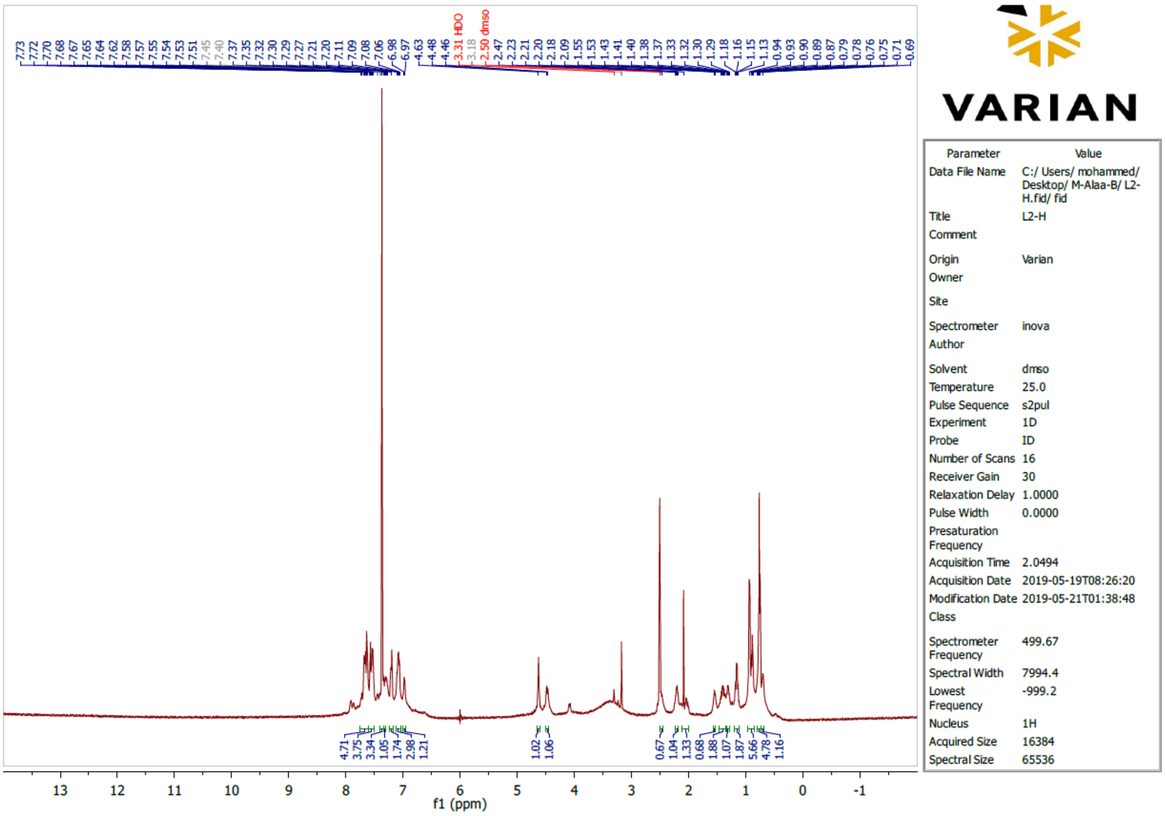

Figure S5. ^{1}H NMR spectrum of **1**.

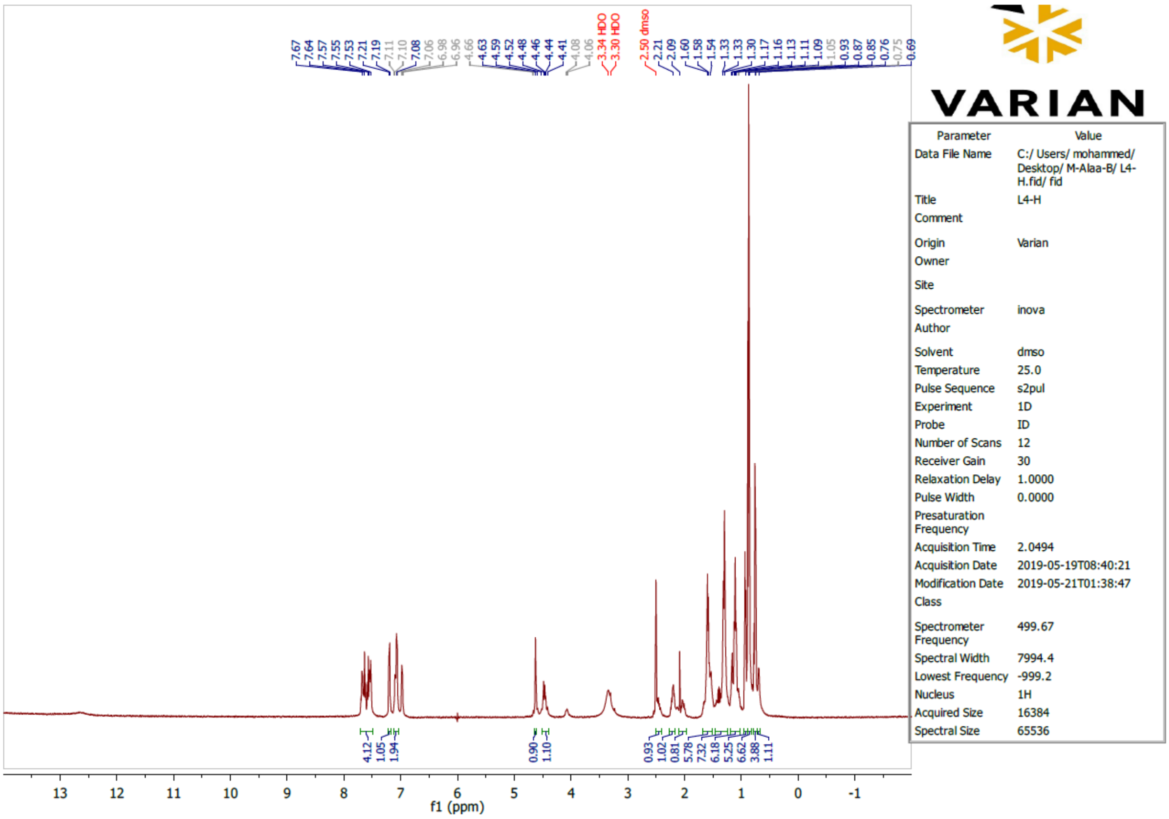

Figure S6. ^{1}H NMR spectrum of **2**.

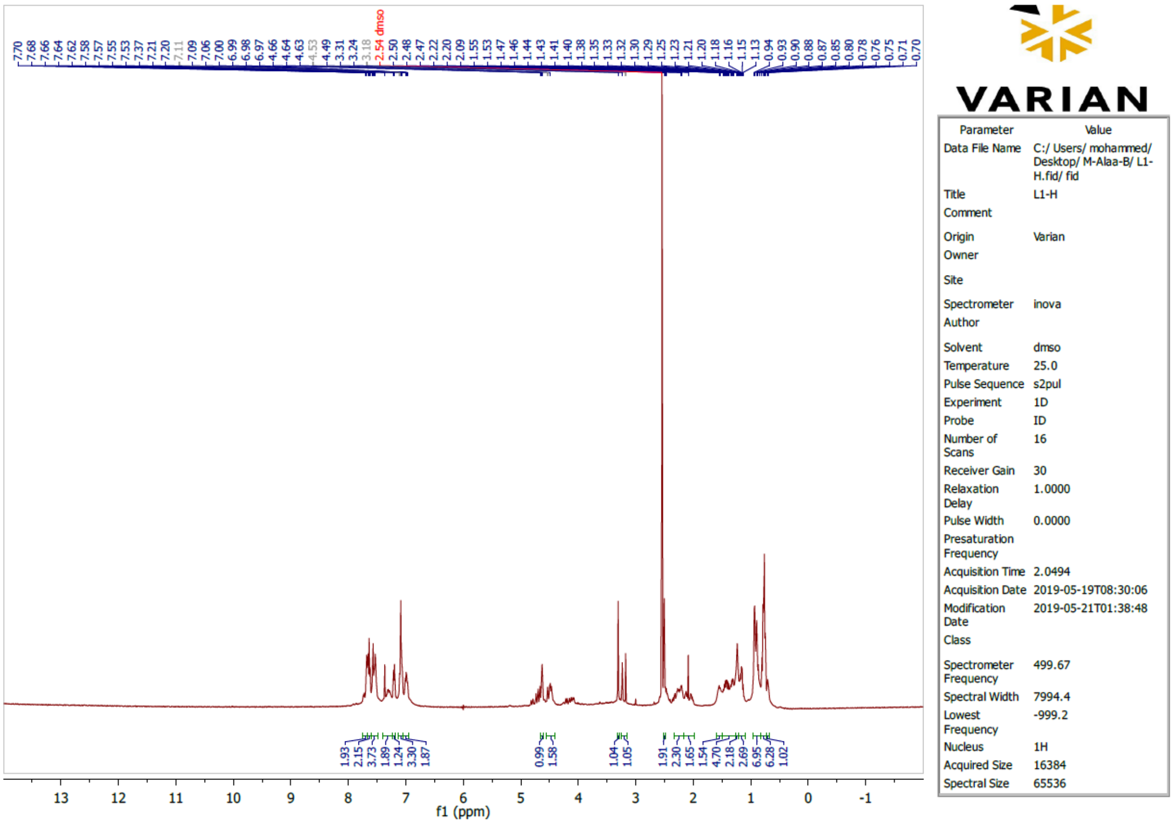

Figure S7. ^{1}H NMR spectrum of **3**.

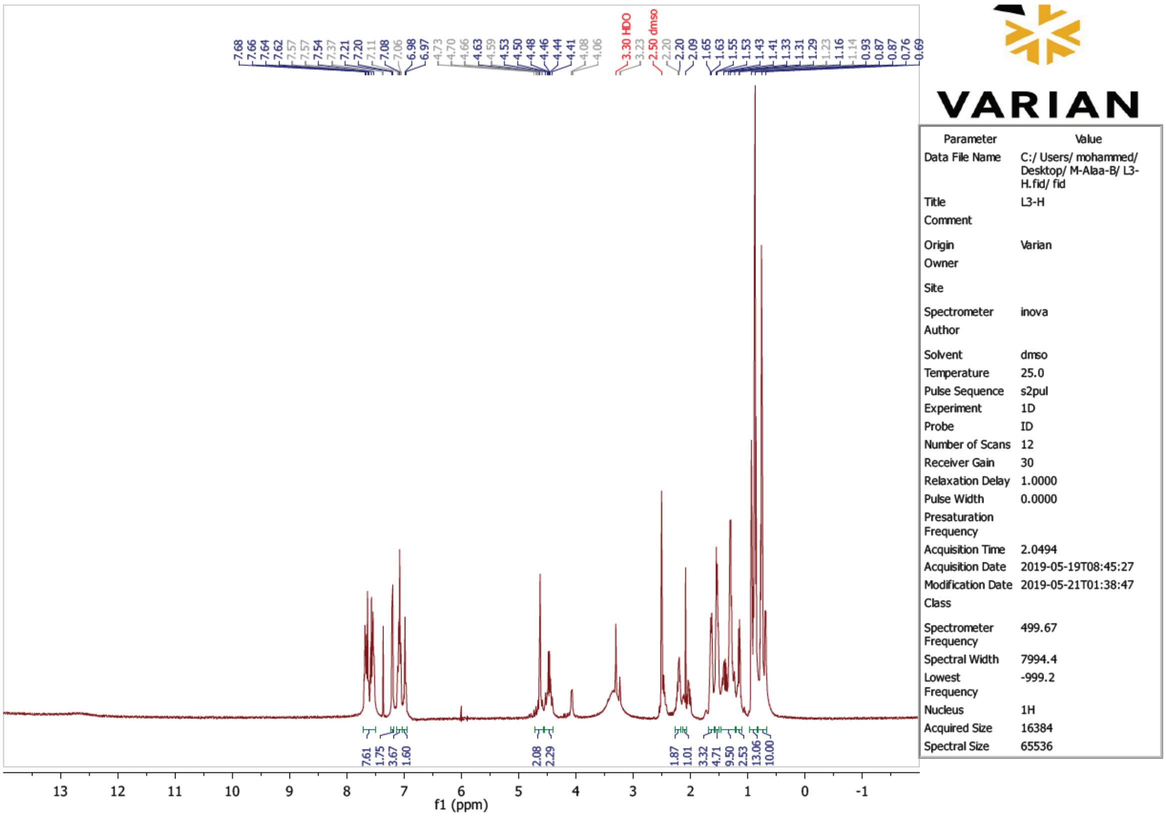

Figure S8. ^{1}H NMR spectrum of **4**.

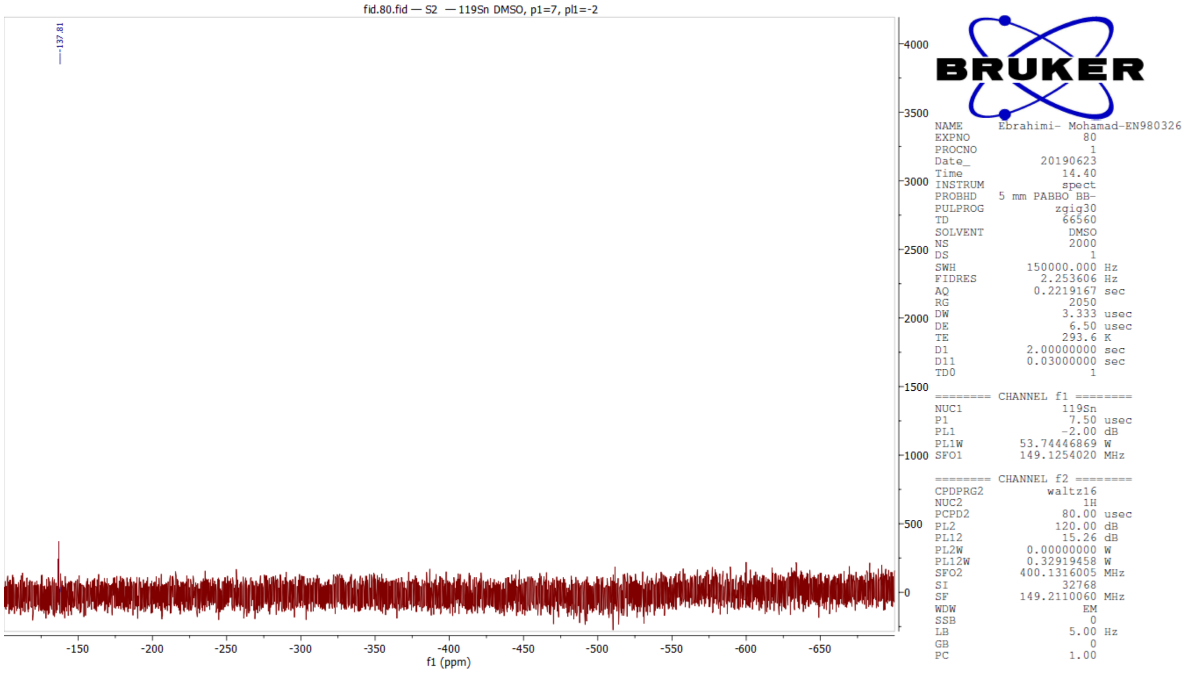

Figure S9. ^{119}Sn NMR spectrum of **1**.

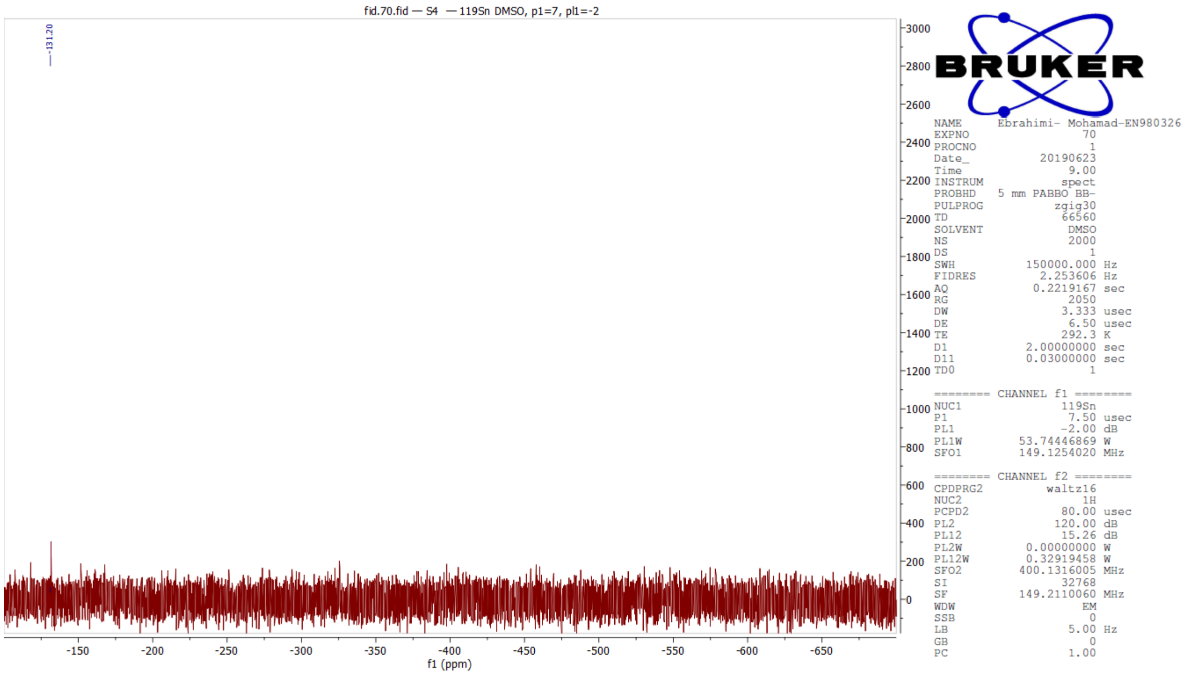

Figure S10. ^{119}Sn NMR spectrum of **2**.

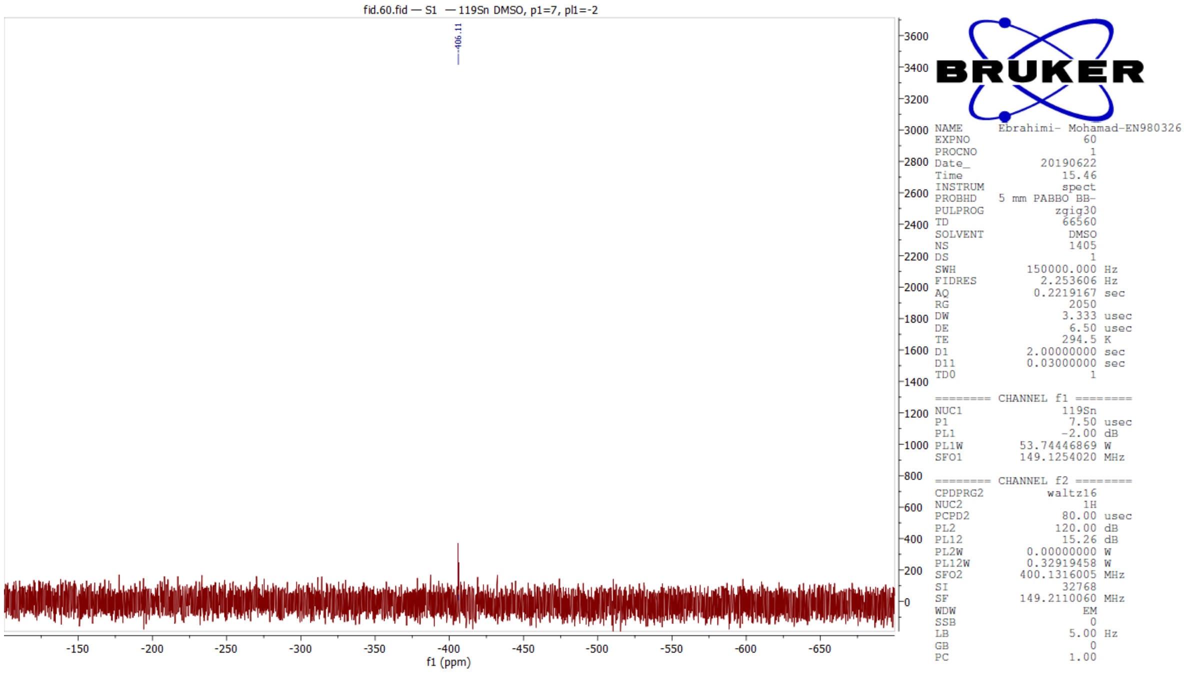

Figure S11. ^{119}Sn NMR spectrum of **3**.

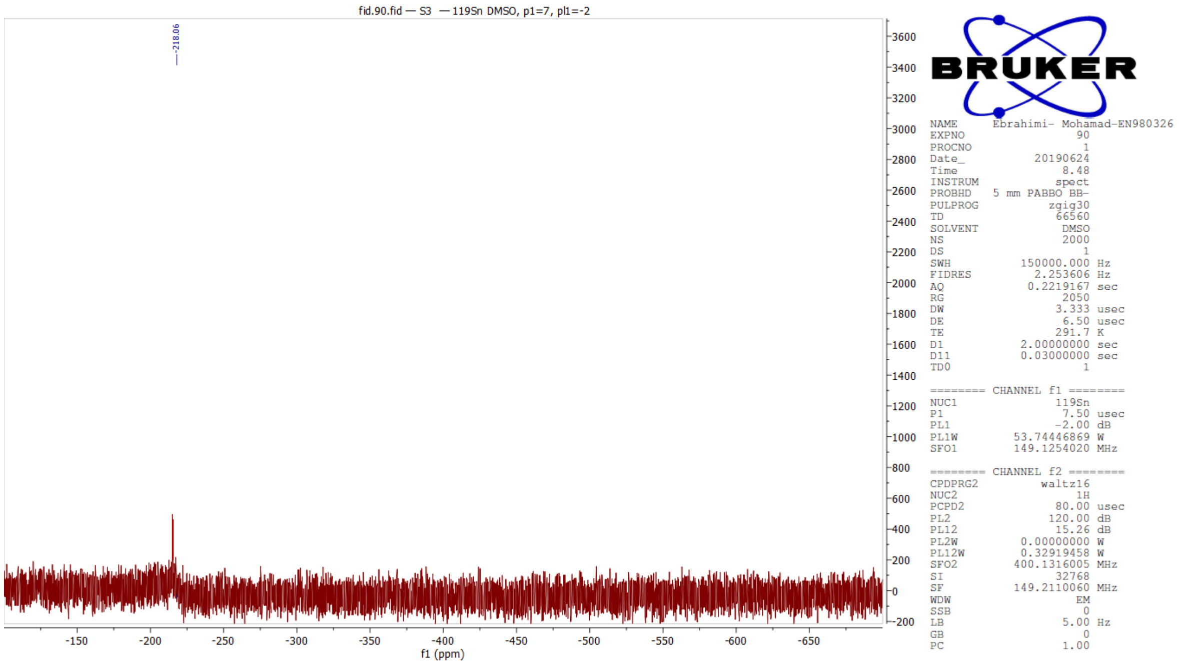

Figure S12. ^{119}Sn NMR spectrum of **4**.

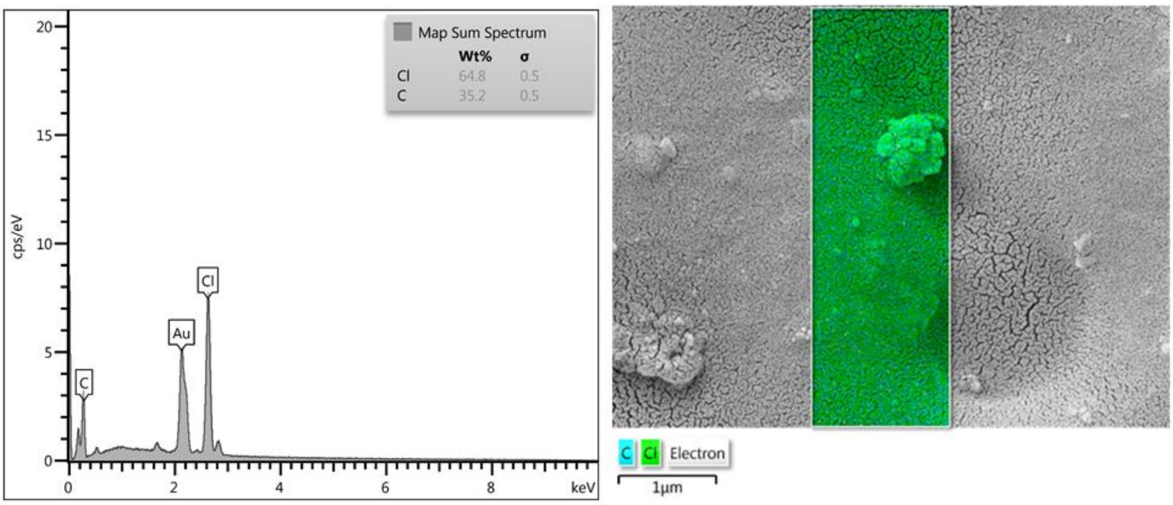

Figure S13. EDX graphs of blank PVC before irradiation

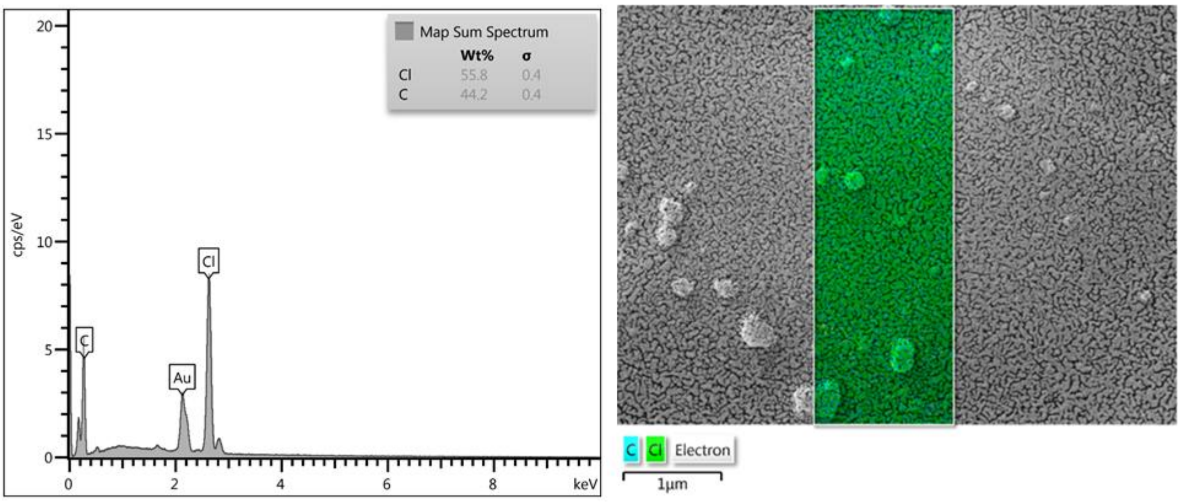

Figure S14. EDX graphs of blank PVC after irradiation.

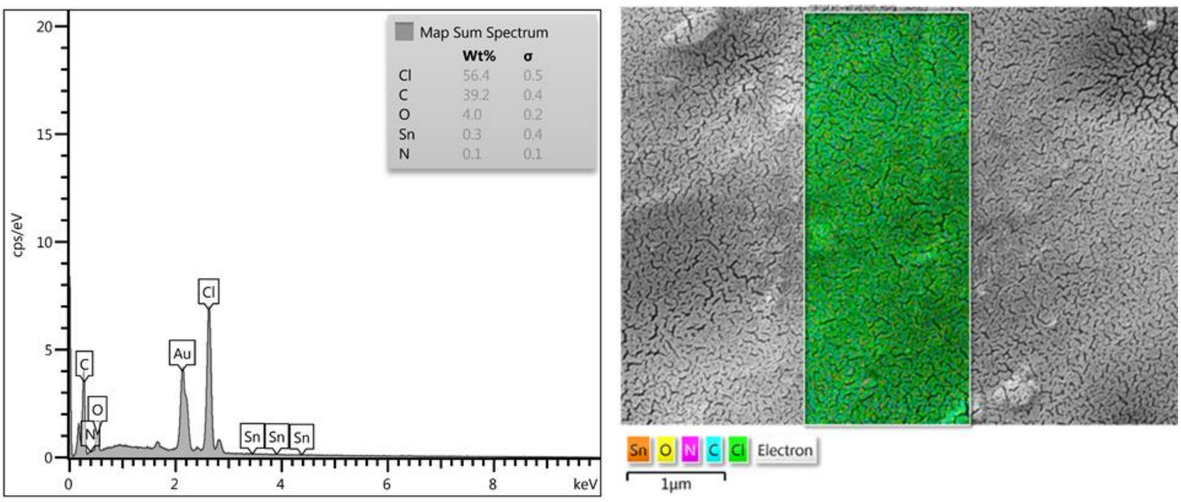

Figure S15. EDX graphs of PVC + complex **1** after irradiation.

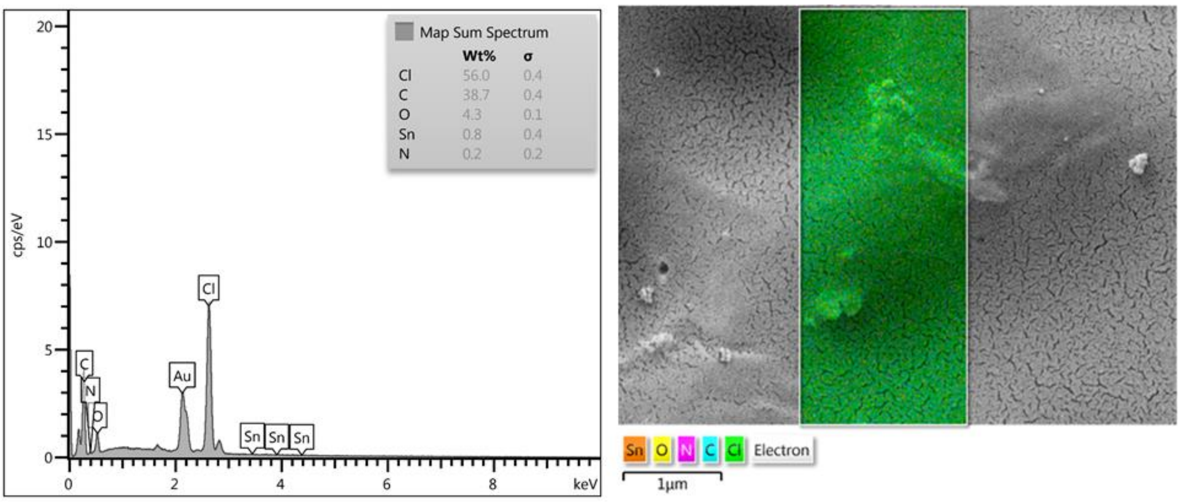

Figure S16. EDX graphs of PVC + complex **2** after irradiation.

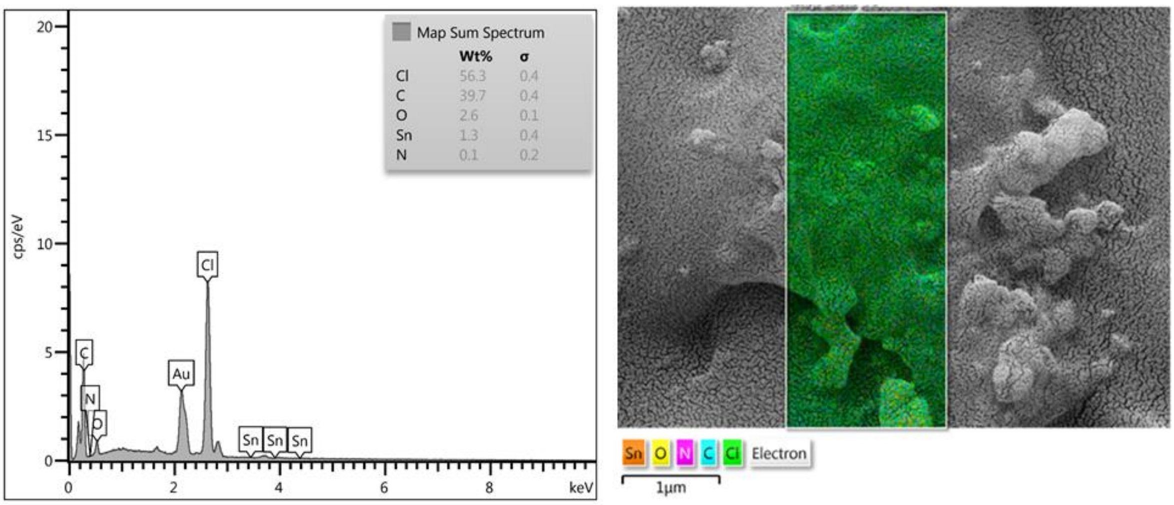

Figure S17. EDX graphs of PVC + complex **3** after irradiation.

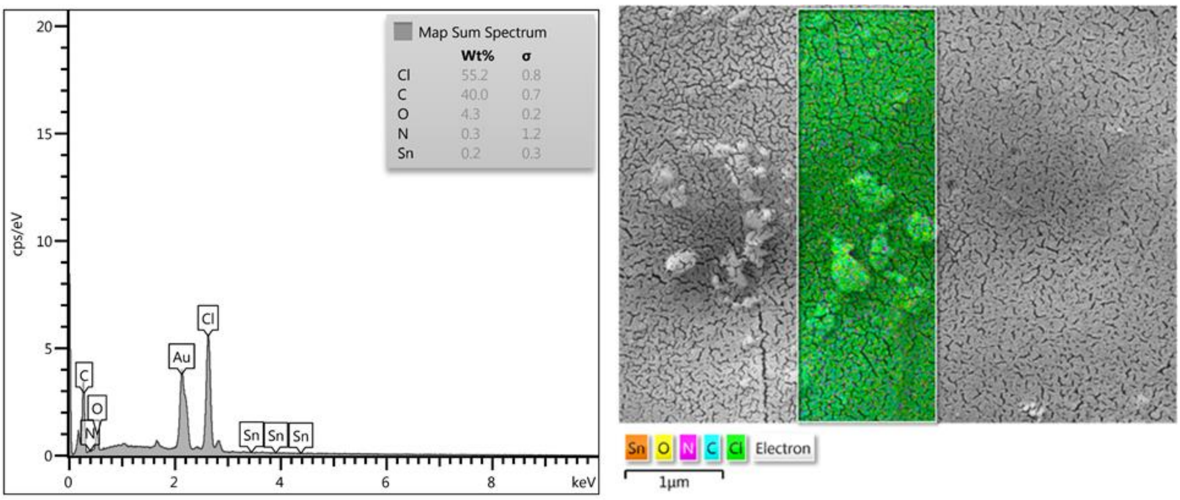

Figure S18. EDX graphs of PVC + complex **4** after irradiation.
